# Physiological mechanisms of *Piriformospora indica*- *Glycyrrhiza Uralensis* Fisch symbiosis in regulating growth and medicinal compound biosynthesis under salt stress

**DOI:** 10.3389/fpls.2026.1802147

**Published:** 2026-05-13

**Authors:** Su-yang Tang, Peng-fei Liu, Chen Fu, Si-ying Tang, Xian-fu Zhou, Bing-gan Lou

**Affiliations:** 1 Ministry of Agriculture and Rural Affairs Key Laboratory of Molecular Biology of Crop Pathogens and Insect Pests, Institute of Biotechnology, Zhejiang University, Hangzhou, China; 2 State Key Laboratory of Soil Pollution Control and Safety, Zhejiang University, Hangzhou, China; 3 Xinjiang Huafang Chinese Herbal Medicine Co., Ltd., Yuli County, Bayingolin Mongol Autonomous Prefecture, Xinjiang Uyghur Autonomous Region, China

**Keywords:** *Glycyrrhiza uralensis* Fisch, *Piriformospora indica*, glycyrrhizic acid, liquiritin, phenotypic characteristics, physiological indicators, salt stress

## Abstract

**Introduction:**

*Glycyrrhiza uralensis* Fisch. is a medicinal plant commonly cultivated in salinized soils, where environmental stress suppresses the accumulation of pharmaceutically active components. To date, only limited studies have examined whether *Piriformospora indica*, a root endophytic fungus with growth-promoting and stress-alleviating properties, can improve the salt tolerance and medicinal quality of *G. uralensis*, particularly at the physiological and transcriptional levels.

**Methods:**

In this study, we successfully established a symbiotic system between *G. uralensis* and *P. indica*. To evaluate responses to salt stress, *P. indica*-inoculated and non-inoculated plants were subjected to NaCl treatments at 0, 100, 200, 300, and 350 mM, with 18 biological replicates per treatment. Colonization by *P. indica* was confirmed through microscopic examination and molecular identification. Growth phenotypes, antioxidant enzyme activities, membrane lipid peroxidation levels, chlorophyll-related indices, and the accumulation of key medicinal components were systema4tically quantified in symbiotic *G. uralensis* across different growth stages.

**Results:**

Inoculation with *P. indica* significantly increased plant height, root length, and dry weight by 27.8%, 25.5%, and 52.2%, respectively. The symbiotic association enhanced the activities of the antioxidant enzymes superoxide dismutase (SOD) and peroxidase (POD) by 48.4% and 27.5%, respectively. Although malondialdehyde (MDA) content initially increased by 16.5% due to early fungal colonization, the canopy SPAD value simultaneously increased by 20.3%. These findings suggest that *P. indica* colonization is associated with differential oxidative stress responses between roots and shoots. Furthermore, under the high salt concentration of 300 mM NaCl, the contents of liquiritin and glycyrrhizic acid were markedly increased by 124.7% and 62.5%, respectively. *P. indica* enhanced the accumulation of secondary metabolites by modulating key rate-limiting enzyme genes rather than indiscriminately activating entire metabolic pathways. For example, the symbiont significantly upregulated *GuHMGR* in triterpenoid saponin biosynthesis and *GuCHR* in flavonoid biosynthesis. These transcriptional changes may contribute to alleviating salt-induced constraints on secondary metabolite accumulation.

**Discussion:**

In conclusion, *P. indica* colonization significantly improved growth performance, stress resistance, and medicinal compound accumulation in *G. uralensis* under salt stress. This study provides a theoretical foundation for improving the cultivation quality of *G. uralensis* in saline soils.

## 1 Introduction

*Glycyrrhiza uralensis* Fisch., a traditional Chinese medicinal herb, is valued primarily for its dried roots and rhizomes. These tissues are rich in bioactive components such as glycyrrhizic acid and liquiritin, which exhibit pharmacological properties including spleen-tonifying, qi-replenishing, heat-clearing, and detoxifying effects (Zhong et al., 2022). It is widely distributed across the arid and semi-arid regions of Eurasia, particularly in Northern China, Mongolia, and Russia (Liu et al., 2013). *G. uralensis* has seen global market demand from pharmaceutical and industrial sectors grow at an annual rate of 8–10% (Zhong et al., 2022; Zhu et al., 2016). However, the primary cultivation regions in the arid and semi-arid zones of Northwest China are increasingly challenged by widespread soil salinization (Liu et al., 2023). High-salinity environments significantly inhibit root development and impede the biosynthesis of key medicinal compounds through three mechanisms: osmotic stress, ion toxicity (Na^+^ accumulation), and oxidative damage (ROS burst). In particular, salt stress typically imposes an early osmotic phase that restricts water uptake and growth, followed by ionic stress associated with excessive Na^+^ accumulation, disruption of K^+^/Na^+^ homeostasis, and accelerated tissue damage (Munns and Tester, 2008). Consequently, the concentrations of liquiritin and glycyrrhizic acid in cultivated plants are significantly lower than those found in wild populations (Yamamoto et al., 2025)。.

*Piriformospora indica* is a broad-spectrum endophytic fungus known to significantly enhance the growth and metabolism of various medicinal plants. Extensive research has demonstrated that *P. indica* improves biomass accumulation, nutrient absorption efficiency, and the synthesis of bioactive compounds in host plants. These benefits are mediated by the regulation of phytohormone balance, expansion of the root absorption surface, activation of antioxidant systems, and the upregulation of key genes involved in secondary metabolic pathways (Lou et al., 2007). For instance, *P. indica* colonization has been shown to double the biomass of *Spilanthes calva* and *Withania somnifera* (Rai et al., 2001). Furthermore, it significantly enhances the accumulation of critical medicinal metabolites, including Bacosides in *Bacopa monnieri*, polysaccharides in *Aloe vera*, and Artemisinin in *Artemisia annua* (Kharde et al., 2023; Rahman et al., 2020). However, the effects reported for *P. indica* are not always uniform and may vary depending on host species, developmental stage, stress intensity, and growth conditions. Salt stress disrupts metabolic homeostasis in medicinal plants, forcing a physiological trade-off where primary growth is suppressed to fuel the accumulation of defensive secondary metabolites against oxidative damage. Consequently, this stress-induced metabolic reprogramming significantly alters the concentration of bioactive compounds, determining the ultimate biomass and medicinal value of the plant (Lou et al., 2007).

Despite these findings, studies specifically addressing whether *P. indica* can establish effective symbiosis with *G. uralensis* remain limited. In addition, the physiological and molecular mechanisms by which this symbiosis may regulate growth and the accumulation of characteristic bioactive compounds under salt stress are still unclear. Focusing on *G. uralensis* as a representative Fabaceae species, this study aims to elucidate the colonization patterns of *P. indica* under salt stress, as well as its mechanisms for promoting growth and enriching medicinal constituents. By demonstrating the dual benefits of improving salt tolerance and promoting secondary metabolism, these findings will provide a theoretical basis for the application of *P. indica* in the high-efficiency cultivation of *G. uralensis* and contribute to the sustainable production of this medicinal resource.

## 2 Materials and methods

### 2.1 Plant materials and fungal strains

#### 2.1.1 Plant and fungal sources

*G. uralensis* seeds used in this study were collected from Northern Shanxi, China. The species was authenticated based on morphological characteristics and ITS sequence alignment. Seeds were stored at 4°C prior to use. The endophytic fungus *P. indica* (*Serendipita indica*) was kindly provided by Professor Ralf Oelmüller (University of Jena, Germany) and stored at -80°C.

#### 2.1.2 Culture media

Media employed in this study included Potato Dextrose Agar (PDA), Potato Dextrose Broth (PDB), and Plant Nutrition Medium (PNM) (Vadassery et al., 2009). All media were prepared according to standard protocols and sterilized by autoclaving at 121°C for 20 min.

### 2.2 Experimental methods

#### 2.2.1 *G. uralensis* cultivation

Plump seeds collected in the current year were surface-sterilized with 70% (v/v) ethanol for 5 min, followed by 1% (w/v) sodium hypochlorite (NaClO) for 5 min with agitation, and rinsed multiple times with sterile distilled water. Seeds were placed on medium containing 1.5% (w/v) agar and incubated in the dark at 25 ± 1 °C for 2–3 days to induce germination. When radicles emerged (1–2 mm), uniformly germinated seeds were selected for the experiment. Seedlings were transplanted into two types of substrates according to the experimental design: (1) native soil collected from the habitat in Xinjiang, and (2) a mixed substrate of Hawita peat soil, horticultural vermiculite, and perlite (4:2:1, v/v/v). The substrate moisture content was adjusted to 60% of the maximum water-holding capacity (Chen et al., 2022). To elucidate the causal influence of indigenous microbial communities on the growth of *G. uralensis*, this study compared plants grown in autoclaved (121 °C, 30 min, 1.05 bar) versus non-sterilized native soil from Xinjiang under identical environmental conditions. This comparison was conducted as a preliminary experiment to evaluate the influence of native microbiota on seedling growth. Unless otherwise stated, the subsequent pot experiments involving *P. indica* inoculation and salt-stress treatments were conducted in the mixed substrate under non-sterile conditions.

#### 2.2.2 *P. indica* culture and inoculum preparation

Frozen *P. indica* stocks were activated on PDA plates at 25-28 °C for 5–10 days until mycelial vitality was optimal. Fresh mycelial plugs (Ø 5 mm) from the colony edge were transferred to PDB and incubated at 25-28 °C with shaking (180 rpm) for 5–10 days. Mycelia were harvested and diluted with the suspension matrix at a ratio of 1:100 (w/w). This suspension was inoculated onto twice-sterilized wheat grain carriers and cultured at 25-28 °C for 5–10 days to produce highly active inoculum (Liu, 2015).

#### 2.2.3 Establishment of *in vitro* co-culture system

*P. indica* mycelial plugs (Ø 5 mm) were placed on PNM medium and pre-cultured at 25-28 °C for 5–10 days. *G. uralensis* seedlings, previously surface-sterilized (75% ethanol for 2 min, 1% NaClO for 5 min, rinsed 3–5 times with sterile water) and germinated under aseptic conditions (soaked in sterile water for 4 h, then cultured on 1.5% water agar for 5 days), were transplanted to the edge of the fungal colony. The co-culture system was maintained at 25-28 °C with a 16 h/8 h light/dark photoperiod to facilitate targeted root colonization.

#### 2.2.4 Establishment of pot co-culture system

During the transplantation of germinated *G. uralensis* into the mixed substrate, wheat grain inoculum containing active *P. indica* mycelia was buried in the rhizosphere zone. A preliminary inoculum-gradient experiment (0, 4, 8, 12, 16, and 20 g per pot) was conducted to optimize the inoculation level, and 12 g per pot was subsequently selected for all formal pot experiments described in this study. Plants were cultivated in a controlled greenhouse environment (26-28 °C) to assess symbiotic effects.

#### 2.2.5 Dual-method identification of *P. indica* colonization

##### 2.2.5.1 Microscopic identification

Colonization was verified using a combination of cryosectioning and Trypan blue staining, modified from Shi (2015).

###### 2.2.5.1.1 Cryosectioning

Root samples collected 30–50 days post-inoculation (dpi) were sequentially infiltrated with 30% sucrose solution (5–6 h), a sucrose-embedding medium mixture (1:1, v/v, 12 h), and pure embedding medium (3–4 h). Sections were mounted in 10% glycerol to preserve mycelial morphology.

###### 2.2.5.1.2 Trypan blue staining

Roots were digested in 10% KOH for 12 h, rinsed with distilled water, neutralized with 1% HCl for 3–5 min, and stained with 0.05% Trypan blue solution (0.4% Bioshark stock diluted in 0.9% NaCl). Colonization structures were observed under an optical microscope at 400 × magnification (Shi, 2015).

##### 2.2.5.2 Molecular identification

Genomic DNA was extracted from the root epidermis of co-cultured plants using a Plant Genomic DNA Extraction Kit (Tiangen Biotech, Beijing). PCR amplification was performed using specific primers for the *P. indica TEF420* gene, with a 20 μL reaction system as described by Shi (2015).

#### 2.2.6 Salt stress treatments

A two-factor nested design was employed: Colonization status (*P. indica*-inoculated vs. non-inoculated) × NaCl concentration (0, 100, 200, 300, and 350 mM). Stress treatments commenced 40 days after the establishment of symbiosis (Li et al., 2022). The experiment consisted of 10 treatment groups (2 × 5), with 18 biological replicates per group. Plants were irrigated with 20 mL of the respective NaCl solution. Salt stress was applied immediately by directly irrigating the pots with 20 mL of the respective NaCl solutions (0, 100, 200, 300, and 350 mM) to achieve target osmotic shock. This approach was adopted to establish clearly separated salinity levels within a defined experimental period, thereby facilitating comparison of physiological and metabolic responses among treatments. Notably, the higher salinity levels (300 and 350 mM NaCl) represented extreme stress conditions that may exceed those commonly encountered in field environments; therefore, the results obtained under these treatments should be interpreted with caution when considering their ecological relevance. However, this treatment regime represents an acute salt challenge rather than the gradual salinization often encountered under field conditions, and this limitation should be considered when interpreting the results. The treatments lasted for 60 days, with the respective NaCl solutions applied every 5 days to maintain stable soil salinity, while additional evaporative water loss was replenished with distilled water as needed to ensure consistent osmotic pressure.

#### 2.2.7 Quantification of growth

Leaf senescence dynamics under salt stress were monitored at 10-day intervals from day 40 to day 100 post-symbiosis establishment. Observations were conducted under standard conditions (PAR: 600 μmol·m^-2^·s^-1^; Temperature: 25 ± 1 °C; RH: 60 ± 5%). Following the method of Lu and Tang (2025), recorded metrics included: (1) Yellowing leaf count (leaves showing ≥ 10% visible chlorosis, excluding natural cotyledon senescence); (2) Abscised leaf count; (3) Leaf damage grade (0: asymptomatic to 4: > 75% yellowing or abscission); and (4) Senescence Index (SI), calculated as:


$$SI\;=\;\left[\sum \;Leaf\;symptom\;grade/\left(4\times Initial\;total\;leaf\;count\right)\right]\;\times \;100+\surd \left(Abscised\;leaf\;count\right)$$

In this modified Senescence Index (SI) formula, a square root transformation (√χ) was applied to the abscised leaf count. This is a standard statistical procedure for count data, which typically follows a Poisson distribution where the variance is proportional to the mean. The square root transformation stabilizes the variance and ensures the data better fulfill the normality and homoscedasticity assumptions required for the subsequent Two-way ANOVA (Ghosh et al., 2022). Furthermore, the constant 100 was introduced only as a scaling term to maintain the index within a positive and easily comparable range across treatments; it does not represent a biologically defined threshold. Therefore, interpretation of SI in this study is based on relative differences among treatments rather than on the absolute value itself.

#### 2.2.8 Phenotypic analysis

Plant height was measured as the vertical distance from the stem base to the apical growth point. Root length was measured from the root collar (rhizome junction) to the tip of the longest root (Chen, 2023). Samples were scanned using an Epson Expression 12000XL scanner (4800 dpi) and analyzed via ImageJ with a precision of ± 0.01 mm.

#### 2.2.9 Multi-dimensional biomass analysis

Biomass was assessed following Gao et al. (2025). Total Fresh Weight (TFW) was recorded within 30 s of harvest. Plants were separated into shoots and roots to determine Shoot Fresh Weight (SFW) and Root Fresh Weight (RFW) (roots were washed and blot-dried). Samples were heat-fixed at 105 °C for 30 min and dried at 55 °C to constant weight to obtain Shoot Dry Weight (SDW) and Root Dry Weight (RDW). Total Dry Weight (TDW) was calculated as the sum of SDW and RDW. The Root Dry Mass Proportion (RDP) was calculated as: RDP= (RDW/TDW) × 100%.

#### 2.2.10 Physiological measurements

Activities of superoxide dismutase (SOD), malondialdehyde (MDA), catalase (CAT), and peroxidase (POD) were determined using assay kits (micro-method, Solarbio Science & Technology, Beijing) and read on a BioTek Synergy H1 microplate reader (Peng et al., 2015).

Proline and soluble sugar (SS) contents were measured using CheKine™ assay kits (Abbkine). Relative chlorophyll content was measured using a SPAD-502Plus meter, and total chlorophyll content was quantified using the A147-1–1 kit (Nanjing Jiancheng Bioengineering Institute), referencing methods by Hui et al. (2014) and Chen et al. (2013). These measurements were used to evaluate chlorophyll status under different treatments and were not intended to represent direct measurements of photosynthetic rate.

#### 2.2.11 HPLC quantification of flavonoids and triterpenoids

Liquiritin and glycyrrhizic acid were quantified using an Agilent 1260 Infinity II LC system equipped with an Agilent InfinityLab Poroshell 120 SB-C18 column (4.6 × 250 mm, 4 μm). The mobile phase consisted of 0.05% phosphoric acid (A) and acetonitrile (B). Conditions were: column temperature 35 °C; flow rate 1.0 mL·min^-1^; injection volume 10 μL; detection wavelength 237 nm. The gradient elution program was: 0–8 min, 81:19 (A:B); 8–35 min, linear gradient to 50:50; 36–40 min, 0:100; 42 min, re-equilibration to 81:19 (Liang et al., 2025; Zhang, 2020).

Standards for liquiritin (A352217) and glycyrrhizic acid (A352214) were obtained from Sangon Biotech (Shanghai). Working solutions were prepared at 95.000 ng·μL^-1^ and 191.050 ng·μL^-1^, respectively.

##### 2.2.11.1 Sample preparation

Dried *G. uralensis* powder (200 mg, passed through a 50-mesh/0.355 mm sieve) was extracted in 100 mL of 70% ethanol via ultrasonication (40 kHz, 200 W) for 30 min at ≤ 25 °C. The extract was centrifuged at 12, 000 × g (4 °C) for 10 min, and the supernatant was filtered through a 0.22 μm filter (Biosharp) prior to analysis.

#### 2.2.12 Gene expression analysis of rate-limiting enzymes

Gene-specific primers (**Table 1**) were designed based on *G. uralensis* transcriptomic data and previous studies (Li et al., 2021; Liu et al., 2012), validated by NCBI Blast alignment.

**Table 1:** Primer sequences employed in this study and functional annotation of their corresponding target genes.

Gene	Enzyme	Function	Primer sequence(5’-3’)	Length (bp)
*GuHMGR*	3-Hydroxy-3-methylglutaryl- CoA reductase	Rate-limiting enzyme for terpenoid precursor synthesis	F:GCCAGCACTAATAGAGGATG R:CTTCAAGGTAGAACTTCAACTG	180 bp
*GuUGAT3*	UDP-Glucuronosyltransferase 3	Final-step enzyme for glycyrrhizic acid synthesis	F:CGGATCATTACAGGAACGA R:AAGCCACTTCAAGCACTC	150 bp
*GuCHR*	Chalcone reductase	Key enzyme in the flavonoid branch pathway	F:GACACTGCTGCTGCTTAT R: GAAGGACAAGATGAGGATGA	170 bp
*GuCHS*	Chalcone synthase	Rate-limiting enzyme for flavonoid synthesis	F: ATGCCTGGTGCTGATTATC R: AATGTGACTGCGGTGATC	160 bp
*Actin*	/	Reference gene	F: GTTGGGATGGGACAGAAGGA R:GGTAAGAAGGACAGGGTGCT	200 bp

Total RNA was extracted using the Easy Plant (Polysaccharide and Polyphenols) RNA Extraction Kit. cDNA was synthesized using the TransScript^®^ Uni All-in-One First-Strand cDNA Synthesis SuperMix for qPCR. Quantitative real-time PCR (qRT-PCR) was performed on a Bio-Rad CFX96 Touch Real-Time PCR Detection System. The 20 μL reaction mixture contained: 3 μL cDNA, 0.4 μL each of forward and reverse primers (10 μM), 10 μL 2×PerfectStart^®^ Green qPCR SuperMix, and 6.2 μL Nuclease-free Water. Cycling conditions were: 94 °C for 30 s; followed by 40 cycles of 94 °C for 5 s, 54 °C for 15 s, and 72 °C for 10 s.

#### 2.2.13 Statistical analysis

Data were processed using SPSS 27.0 and GraphPad Prism 10. All quantitative results are presented as Mean ± Standard Error (SE). One-way ANOVA was used to compare individual treatment combinations within a given experiment, whereas Two-way ANOVA was used for the factorial datasets to assess the main effects of *P. indica* colonization (C), salt concentration (S), and their interaction (C × S). Specifically, data generated under the salt factorial design, including physiological traits, antioxidant enzyme activities, osmotic regulation indices, phytochemical traits, and gene expression profiles, were interpreted primarily on the basis of the Two-way ANOVA, while one-way ANOVA was applied as a complementary analysis for comparisons among treatment means within individual datasets. Growth, physiological and phytochemical data were analyzed via one-way ANOVA using SPSS 27.0, and significant differences between means were determined by LSD (for growth indices) or Duncan’s multiple range tests (for physiological and phytochemical indices) (*P* < 0.01) when variances were homogeneous. The distinction between LSD and Duncan’s multiple range test was retained for consistency with the original data-processing workflow: LSD was applied to growth indices with relatively fewer direct comparisons, whereas Duncan’s multiple range test was used for physiological and phytochemical indices involving broader multi-group comparisons. Phytochemical patterns were visualized using heatmaps in Origin 2025. Chromatographic fingerprint consistency was evaluated using the “Similarity Evaluation System for Chromatographic Fingerprint of Traditional Chinese Medicine” in strict accordance with national standards. Multivariate analysis and model fitting were conducted using SIMCA 14.1 and GraphPad Prism 10, respectively. Additionally, to rigorously evaluate the interactive effects of the experimental treatments, data from the two-factor designs—encompassing physiological traits, antioxidant enzyme activities, osmotic regulation, and gene expression profiles—were further analyzed using a Two-way ANOVA. This analysis explicitly assessed the main effects of *P. indica* colonization (C), salt concentration (S), and their interaction (C × S). It should be noted that differences between sterilized and non-sterilized soil may reflect the influence of background microbial communities, which could interact with *P. indica*. Therefore, the observed effects cannot be attributed exclusively to *P. indica*, and potential microbial interactions should be taken into account when interpreting these results.

## 3 Results

### 3.1 Construction of the *G. uralensis*–*P. indica* symbiotic system

#### 3.1.1 Effects of soil sterilization on seedling growth and symbiosis establishment

The results indicated that plants grown in soil retaining the indigenous microbiota exhibited a significant growth response. Compared to the sterilized soil group, plants in the non-sterilized group showed a 23.9% increase in plant height and a 39.6% increase in root length. Furthermore, total biomass in the non-sterilized group increased by 16.9%, driven primarily by a 54.5% increase in root biomass, while shoot biomass showed no significant difference (**Table 2**). These results suggest that native soil microbiota may influence early seedling growth. Therefore, in the subsequent experiments, the effects attributed to *P. indica* were interpreted within the controlled inoculation framework established in the mixed-substrate pot system, rather than being attributed solely on the basis of the preliminary native-soil comparison.

**Table 2:** Effects of native-soil sterilization on growth performance of *G. uralensis* seedlings.

Treatment	Plant height (cm)	Root length (cm)	Total biomass (g)	Shoot biomass (g)	Root biomass (g)
Non-sterilized	8.77 ± 0.27 ^**^	11.35 ± 0.60 ^**^	0.90 ± 0.01 ^*^	0.61 ± 0.03 ^ns^	0.34 ± 0.03 ^*^
Sterilized	7.08 ± 0.13	8.13 ± 0.29	0.77 ± 0.04	0.50 ± 0.03	0.22 ± 0.02

**P <no><</no> 0.01, *P <no><</no> 0.05, ns, no significant difference; data are from three biological replicates (n = 10 plants per replicate).

#### 3.1.2 Observation of *P. indica* colonization in *G. uralensis* roots

**Figure 1:**
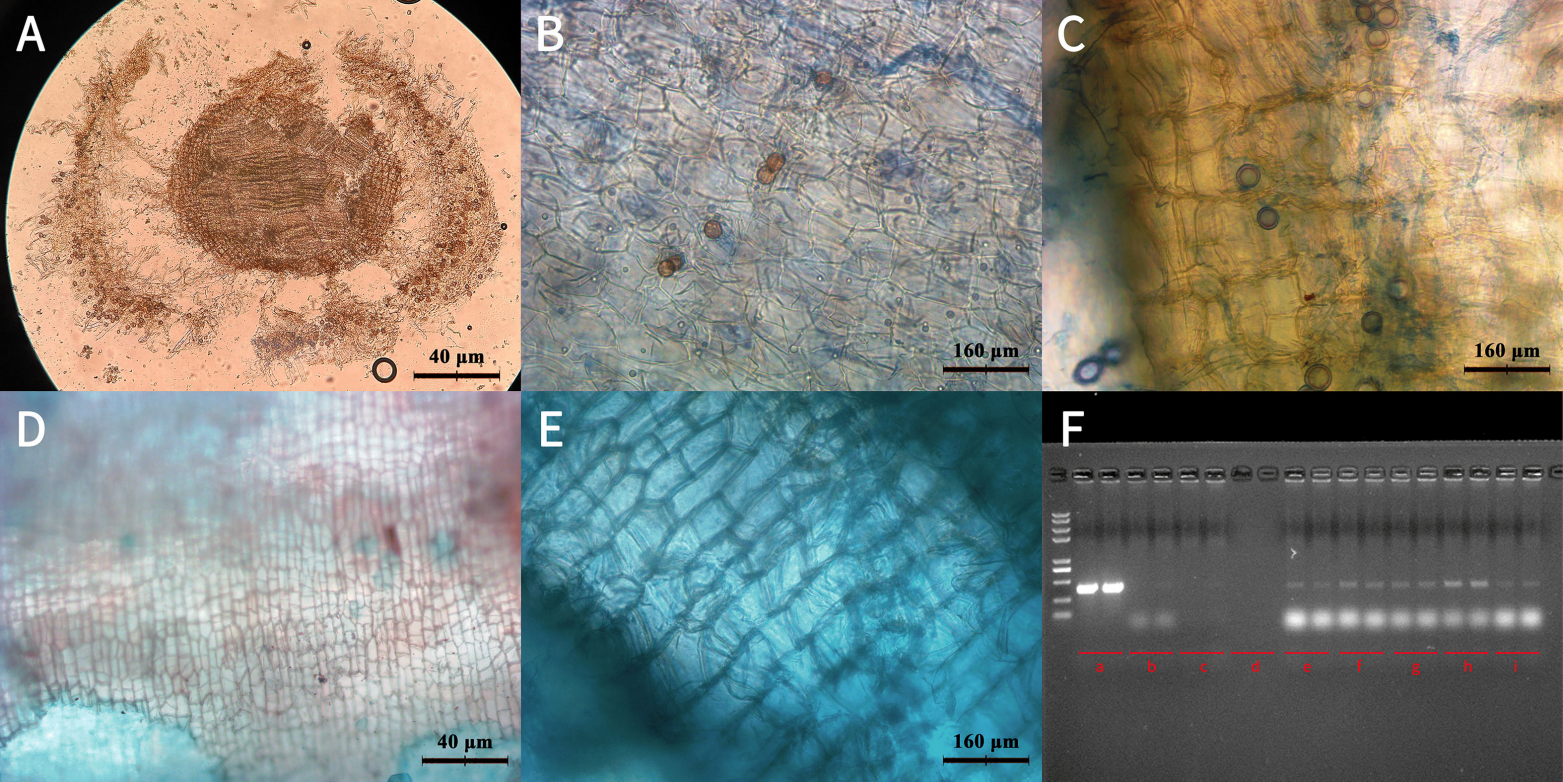
Colonization of *P. indica* in the root system of *G. uralensis.* Notes: **(A)** Cross-section of the maturation zone of licorice (*G. uralensis*) root in the inoculated group at 100× magnification (scale bar = 40 μm). **(B)** Same tissue as in A at 400× magnification (scale bar = 160 μm). **(C)** Chlamydospore clusters in a longitudinal section of the mature root zone of *G. uralensis* from the inoculated group (scale bar = 160 μm). **(D)** Cross-section of the maturation zone of licorice root in the non-inoculated (control) group at 100× magnification (scale bar = 40 μm). **(E)** Same tissue as in D at 400× magnification (scale bar =40μm). **(F)** Temporal PCR amplification profile of the *P. indica TEF420* gene. M: DL2000 DNA marker; lane a, positive control—genomic DNA from *P. indica* pure culture; lane b, negative control—DNA from non-inoculated licorice root; lane c, blank control—H_2_ lane d, empty lane; lanes e–i, samples collected at 30, 35, 40, 45, and 50 days post-inoculation, respectively.Molecular identification using a specific detection system for the P. indica translation elongation factor gene (TEF420) confirmed continuous colonization. Time-series PCR amplification of root DNA detected the specific 420 bp band at all sampled time points (30–50 days) post-inoculation **(F)**, demonstrating stable long-term colonization in the G. uralensis root system.

Microscopic examination was conducted 40 days post-inoculation (dpi). Observations at 100× (**Figures 1A, D**) and 400× (**Figures 1B, C, E**) magnification revealed numerous chlamydospores within the epidermal and cortical intercellular spaces of the root maturation zone in inoculated plants (**Figures 1A–C**). In contrast, no fungal structures were observed in the control group (**Figures 1D, E**). Agarose gel electrophoresis further confirmed the temporal amplification of the *P. indica* TEF420 gene (**Figure 1F**). These findings confirm that *P. indica* successfully colonized the intercellular spaces of the root system.

### 3.2 Growth-promoting effects of *P. indica* colonization on phenotype and biomass accumulation

Inoculation with *P. indica* significantly altered the growth dynamics of *G. uralensis*, and the observed “phasic enhancement” is used here as a descriptive summary of the temporal pattern rather than as a statistically tested stage-specific mechanism (**Table 3**). In the early symbiotic stage (after 45 days of symbiosis), *P. indica* preferentially promoted shoot elongation, increasing plant height by 27.8% compared to controls. During the critical window period (after 50 days of symbiosis), the focus shifted to root expansion; root length increased by 25.5%, driving a peak in fresh weight accumulation (+26.7%) and suggesting improved root growth and biomass accumulation during this period. In the later symbiotic stage (after 55 days of symbiosis), a greater increase in dry matter accumulation was observed. The dry weight increased by 52.2%, which was 3.3 times the rate of fresh weight increase (15.6%), suggesting enhanced biomass deposition under colonization. Temporal differences among these stages were not compared by a dedicated repeated-measures or time-series statistical model, and therefore these temporal patterns should be interpreted with caution.

**Table 3:** Temporal effects of *P. indica* on growth of *G. uralensis.*.

Seedling age	Treatment	Plant height (cm)	Root length (cm)	Dry weight (g)	Fresh weight (g)
45d	*P. indica*	4.46 ± 0.20 **	8.67 ± 0.35 **	0.07 ± 0.01 ns	0.92 ± 0.05 *
	CK	3.49 ± 0.16	6.56 ± 0.41	0.06 ± 0.01	0.76 ± 0.04
50d	*P. indica*	7.49 ± 0.21 **	8.80 ± 0.23 **	0.20 ± 0.01 ns	1.14 ± 0.05 *
	CK	4.86 ± 0.33	7.01 ± 0.37	0.14 ± 0.02	0.90 ± 0.03
55d	*P. indica*	9.87 ± 0.23 **	9.07 ± 0.31 **	0.35 ± 0.02 **	2.07 ± 0.04 *
	CK	8.95 ± 0.25	7.36 ± 0.28	0.23 ± 0.01	1.79 ± 0.08

**P < 0.01, *P < 0.05, ns (not significant); n = 3 biological replicates with 10 plants each. CK denotes the control group without *P. indica* inoculation; *P. indica* denotes the group inoculated with *P. indica*.

### 3.3 Specific activation of antioxidant defense systems and response to oxidative damage

**Figure 2:**
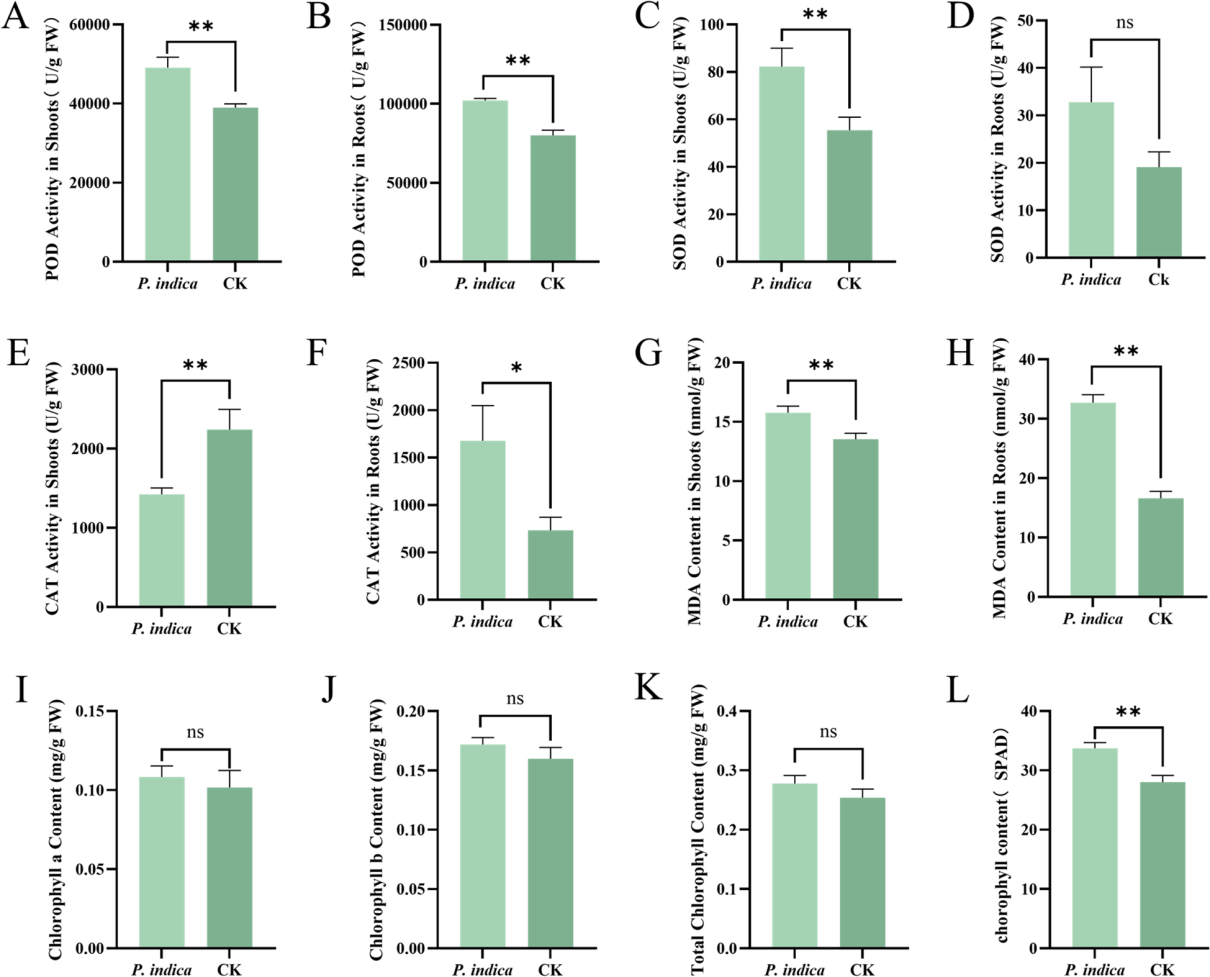
Effects of *P. indica* colonization on physiological indices of *G. uralensis.* Note: **(A)** Effect of *P. indica* on POD content in the shoot of *G. uralensis*. **(B)** Effect of *P. indica* on POD content in the root of *G. uralensis*. **(C)** Effect of *P. indica* on SOD content in the shoot of *G. uralensis*. **(D)** Effect of *P. indica* on SOD content in the root of *G. uralensis*. **(E)** Effect of *P. indica* on CAT content in the shoot of *G. uralensis*. **(F)** Effect of *P. indica* on CAT content in the root of *G. uralensis*. **(G)** Effect of *P. indica* on MDA content in the shoot of *G. uralensis*. **(H)** Effect of *P. indica* on MDA content in the root of *G. uralensis*. **(I)** Effect of *P. indica* on chlorophyll a content in *G. uralensis*. **(J)** Effect of *P. indica* on chlorophyll b content in *G. uralensis*. **(K)** Effect of *P. indica* on total chlorophyll content in *G. uralensis.* **(L)** Effect of *P. indica* on SPAD value in *G. uralensis*. **P < 0.01, *P < 0.05, ns not significant; n = 3 biological replicates, with 10 plants per replicate.

*P. indica* colonization significantly modulated the physiological status of *G. uralensis* (**Figure 2**). In inoculated plants, root peroxidase (POD) activity reached 102, 002 U·g^-1^ FW (**Figure 2B**), significantly higher (27.5%) than the control group’s 80, 032 U·g^-1^ FW. Similarly, shoot POD activity increased by 26.0% to 49, 087 U·g^-1^ FW (**Figure 2A**). Shoot superoxide dismutase (SOD) activity also rose significantly from 55.42 to 82.27 U·g^-1^ FW, an increase of 48.4% (**Figure 2C**). Conversely, shoot catalase (CAT) activity decreased by 36.5% compared to controls (1, 424 vs. 2, 242 U·g^-1^ FW) (**Figure 2E**).

Colonization triggered a specific pattern of membrane lipid peroxidation characterized by an organ-specific gradient. Root malondialdehyde (MDA) content reached 32.7 nmol·g^-1^ FW, a 97.0% increase over the control (**Figure 2H**), which may be associated with oxidative processes accompanying early fungal colonization, although this should not be interpreted directly as a necessary cost of symbiosis establishment without further evidence. In contrast, shoot MDA content (15.78 nmol·g^-1^ FW) showed only a mild 16.5% elevation compared to the control (13.54 nmol·g^-1^ FW) (**Figure 2G**). These results suggest an organ-differentiated oxidative response between roots and shoots; however, whether ROS were maintained within a strictly physiological range was not directly demonstrated in this study.

### 3.4 Adaptive enhancement of photosynthetic performance under stress

Colonization by *P. indica* induced an adaptive enhancement in chlorophyll-related status. The SPAD value in the inoculated group reached 33.72, a 20.3% increase over the control (28.02, *P* < 0.01) (**Figure 2L**), indicating increased chlorophyll density. Concurrently, the chlorophyll a/b ratio rose by 6.8% (**Figures 2I, J**), suggesting a potential adjustment in the chlorophyll composition under colonization. Because gas-exchange and chlorophyll-fluorescence measurements were not performed, these results are interpreted as indirect evidence of chlorophyll status rather than direct evidence of improved photosynthetic efficiency.

### 3.5 Quantification of growth dynamics under salt stress

The temporal evolution of the Senescence Index (SI) over the full cycle (**Table 4**) exhibited a three-stage escalation pattern: a latent phase (0–60 days, SI = 0), an initiation phase (60–80 days, SI rising with salt concentration), and an outbreak phase (80–100 days, accelerated SI growth in high-salt groups). To rigorously evaluate this temporal progression, a Two-way ANOVA was applied across the growth cycle.

**Table 4:** Full-cycle temporal SI dynamic matrix.

Salt concentration	Treatment	30d SI ± SE	60 d SI ± SE	70 d SI ± SE	80 d SI ± SE	90 d SI ± SE	100 d SI ± SE
0 mM	CK	0	2.08 ± 2.41	6.48 ± 7.48	18.89 ± 2.10	27.19 ± 3.37	44.17 ± 3.99
	*P. indica*	0	1.97 ± 0.99	3.44 ± 1.06	4.92 ± 1.50	10.61 ± 2.37	17.24 ± 3.01
100 mM	CK	0	4.76 ± 4.77	10.21 ± 5.94	16.90 ± 2.19	26.71 ± 0.91	41.63 ± 5.32
	*P. indica*	0	3.01 ± 0.12	5.19 ± 1.45	9.99 ± 1.25	12.37 ± 0.69	22.24 ± 2.50
200 mM	CK	0	7.59 ± 4.32	9.49 ± 5.14	15.14 ± 7.98	30.63 ± 4.14	51.89 ± 9.32
	*P. indica*	0	2.90 ± 1.60	4.33 ± 1.46	7.37 ± 1.94	22.67 ± 2.52	36.17 ± 3.14
300 mM	CK	0	9.39 ± 3.04	9.83 ± 5.29	25.04 ± 6.12	43.10 ± 1.51	68.33 ± 3.60
	*P. indica*	0	4.47 ± 2.46	9.06 ± 1.76	17.50 ± 0.58	28.94 ± 1.64	43.33 ± 5.16
350 mM	CK	0	10.71 ± 2.68	12.50 ± 1.00	28.99 ± 3.33	48.43 ± 1.80	99.14 ± 4.71
	*P. indica*	0	7.87 ± 3.95	10.83 ± 1.60	14.67 ± 1.50	28.76 ± 1.27	37.11 ± 4.58

CK denotes the control group without *P. indica* inoculation; *P. indica* denotes the group inoculated with *P. indica*. Furthermore, Two-way ANOVA was conducted to assess the temporal progression. While main effects (Salt and Colonization) became significant from day 80 (*P* < 0.001), a significant interaction (S × C) only emerged at day 100 (*P* < 0.001), indicating a time-dependent interaction at the terminal stage rather than a continuous synergistic effect across all stages.

During the initial latent phase (60–70 days), neither salt concentration nor *P. indica* colonization significantly impacted the SI, indicating an early stress acclimation period. As the stress duration extended into the initiation phase (80–90 days), both salt stress and fungal colonization emerged as highly significant independent factors (main effects: *P* < 0.001). During this period, no significant interaction was detected (S × C: ns), demonstrating that *P. indica* provided a consistent, additive reduction in senescence.

At the terminal stage (100 days), a highly significant interaction between salt stress and fungal colonization was detected (S × C: *P* < 0.001). In the non-inoculated group (C), exposure to 350 mM NaCl resulted in a 100-day SI of 99.14 ± 4.71, a 124.4% increase compared to the 0 mM group, confirming that prolonged high salt drives severe senescence. However, the inoculated group (P) showed a significantly lower SI under these extreme conditions. At 350 mM, the 100-day SI of the group (P) (37.11 ± 4.58) was significantly restricted to only 37.4% of that in the group (C). Because earlier stages showed no significant interaction, this result is interpreted as a time-dependent statistical emergence of interaction at the terminal stage, rather than as evidence for a uniformly synergistic effect throughout the entire stress period. Accordingly, the protective effect of *P. indica* under extreme salt stress was most pronounced at 100 days, with a maximum reduction of 62.6% relative to the non-inoculated group (**Table 4**).

### 3.6 *P. indica* enhances salt tolerance in *G. uralensis*

#### 3.6.1 Restructuring of SOD response patterns

**Figure 3:**
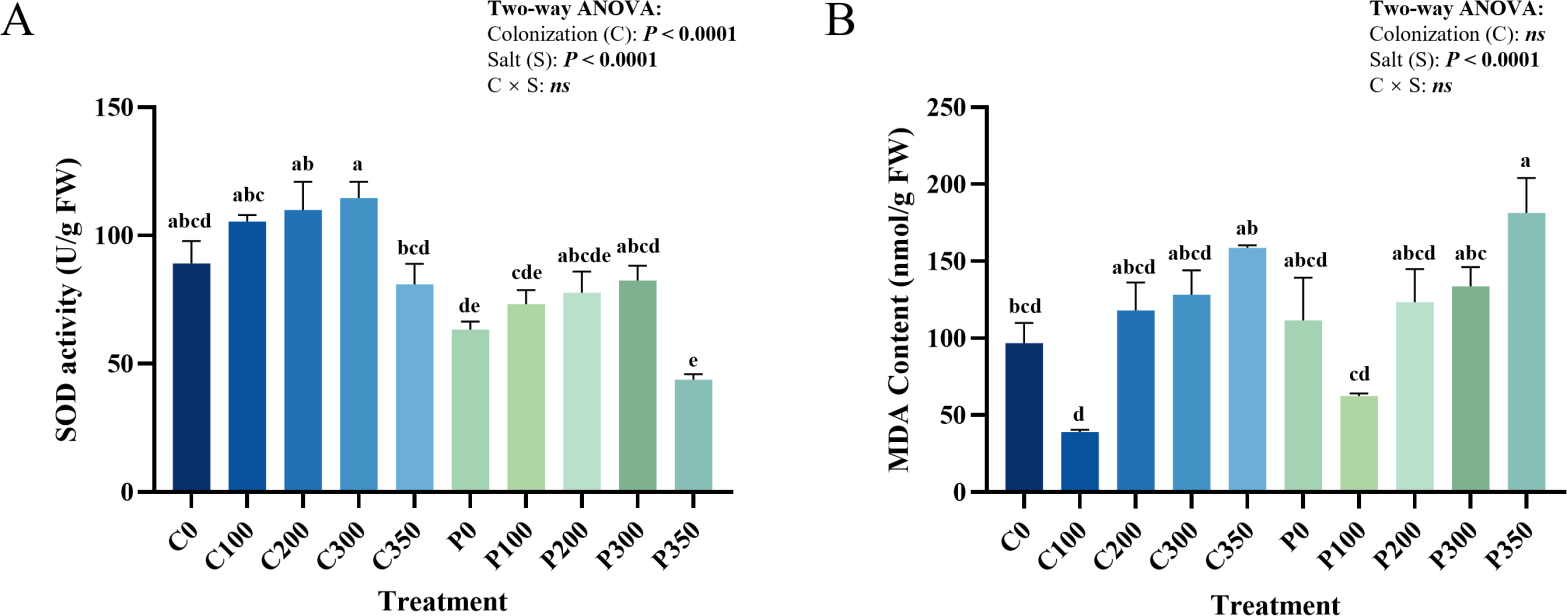
Impacts of salt stress and *P. indica* inoculation on the physiological indices of *G. uralensis.* **(A)**, SOD activity; **(B)**, MDA content. C denotes the control group without *P. indica* inoculation; P denotes the group inoculated with *P. indica*. Different lowercase letters indicate significant differences among all treatment combinations based on a One-way ANOVA followed by Duncan’s multiple range test (*P* < 0.05). The embedded text panels display the *P*-values derived from the Two-way ANOVA, which rigorously assesses the main effects of *P. indica* colonization (C), salt stress (S), and their interaction (C × S).

To rigorously evaluate the antioxidant responses, a Two-way ANOVA was applied to the SOD activity data (**Figure 3A**). Both *P. indica* colonization and salt concentration exerted significant independent main effects (P < 0.01 for both), with no significant interaction detected (C × S: ns). Under salt stress, SOD activity in the uninoculated CK group peaked at 300 mM (114.60 ± 6.32 U·g^-1^ FW, + 28.6% vs. 0 mM) but collapsed under severe stress (350 mM), dropping significantly by 29.4% (80.97 ± 8.02 U·g^-1^ FW). Conversely, while the inoculated *P. indica* group showed lower baseline activity at 300 mM (72.0% of the CK group), it maintained high stability at 350 mM (standard error reduced by 73.0%). This pattern indicates that *P. indica* colonization and salt stress exerted largely additive effects on SOD activity across the salt gradient, rather than reflecting a significant interaction-driven buffering mechanism.

#### 3.6.2 Systemic inhibition of MDA accumulation

For MDA accumulation (**Figure 3B**), the Two-way ANOVA revealed that the variance was predominantly driven by the severity of the salt stress (S: *P* < 0.001), without a significant global interaction (C × S: ns). However, targeted physiological protection was observed at specific stress levels. In the CK group, MDA levels dipped at 100 mM (62.46 ± 1.69 nmol·g^-1^ FW, -44.0% vs. control) but rose linearly at 200 mM, surging to a peak of 181.4 ± 22.67 nmol·g^-1^ FW at 350 mM (+45.3% vs. control). Inoculation effectively suppressed this extreme oxidative damage; excluding the 0 mM condition, MDA levels in the *P. indica* group were generally lower than in the CK group. Specifically, at 100 mM, MDA in the P group was 37.3% lower than in the CK group. At 350 mM, MDA (158.60 ± 1.64 nmol·g^-1^ FW) was significantly restricted compared to the CK group, accompanied by a 92.8% reduction in standard error, indicating reduced membrane oxidative damage under inoculation at high salinity.

#### 3.6.3 Coordinated SOD-MDA regulation mechanism

Given the lack of a statistical interaction (C × S: ns) in both SOD and MDA responses, the relationship between these two markers should be interpreted cautiously. These results indicate that *P. indica* colonization and salt stress exert largely additive effects on plant physiological responses, rather than coordinated regulation. Under low-to-moderate salt stress (100–200 mM), the *P. indica* group prioritized MDA suppression (39.15 ± 1.32 nmol·g^-1^ FW at 100 mM) without a corresponding increase in SOD activity, which remained at 69.3% of the CK group level. Under high salt stress (≥ 300 mM), the *P. indica* group achieved a 12.6% reduction in MDA damage (at 350 mM) while utilizing only 54.1% of the SOD activity found in controls. The synchronized reduction in variability for both markers suggests a context-dependent physiological response under salt stress conditions, rather than a coordinated optimization mechanism. However, because no direct measurements of energy or carbon cost were performed, the “low-cost, high-efficiency” interpretation should be regarded as a working hypothesis rather than a confirmed mechanism.

### 3.7 Expression analysis of key genes in flavonoid and saponin biosynthesis

#### 3.7.1 Dynamic response of liquiritin accumulation

To rigorously evaluate the accumulation of medicinal components, a Two-way ANOVA was applied. For liquiritin content (**Table 5**), both salt stress and *P. indica* colonization exerted highly significant independent main effects (*P* < 0.001), with no significant interaction detected (C × S: *P* = 0.074). This statistical pattern indicates a robust additive enhancement rather than a synergistic over-accumulation.

**Table 5:** Impact of *P. indica* on the accumulation of medicinal constituents in *G. uralensis* under salt stress.

Salt concentration	Treatment	Liquiritin content (%)	Glycyrrhizic acid content (%)
0 mM	CK	0.167 ± 0.023 c	1.372 ± 0.161 c
	*P. indica*	0.422 ± 0.034 a	2.421 ± 0.231 ab
100 mM	CK	0.258 ± 0.018 bc	1.720 ± 0.209 ac
	*P. indica*	0.443 ± 0.044 a	2.510 ± 0.226 a
200 mM	CK	0.193 ± 0.023 c	1.545 ± 0.162 c
	*P. indica*	0.368 ± 0.041 ab	2.054 ± 0.146 ac
300 mM	CK	0.220 ± 0.011 bc	1.590 ± 0.212 bc
	*P. indica*	0.494 ± 0.031 a	2.583 ± 0.118 a
350 mM	CK	0.128 ± 0.010 c	1.310 ± 0.110 c
	*P. indica*	0.259 ± 0.027 bc	1.888 ± 0.134 ac

CK denotes the control group without *P. indica* inoculation; *P. indica* denotes the group inoculated with *P. indica*. Additionally, Two-way ANOVA confirmed significant main effects of both salt stress and *P. indica* colonization on liquiritin and glycyrrhizic acid contents (*P* < 0.05), with no significant interaction detected (C × S: ns), reflecting a stable additive enhancement of medicinal constituents.

Different lowercase letters indicate significant differences among treatments at p < 0.05.

Based on the quantitative analysis of liquiritin (**Table 5**), *P. indica* inoculation induced a salt-concentration-dependent differential accumulation pattern. In the non-inoculated group (C), liquiritin accumulation followed a “rise-then-fall” stress response pattern. Levels peaked at 100 mM salt concentration (0.2583 ± 0.0178 mg·g^-1^), representing a significant 55.0% increase over the control (0 mM), suggesting an association between mild salt stress and increased flavonoid glycoside accumulation. However, when salt concentration rose to 350 mM, the content dropped sharply to 0.1275 ± 0.0101 mg·g^-1^, a 50.6% decrease from the peak, consistent with a strong inhibitory effect of severe salt stress on flavonoid glycoside accumulation.

*P. indica* colonization significantly restructured the liquiritin accumulation pattern. The inoculated group (P) maintained higher accumulation levels across all salt concentrations, reaching a maximum at the critical threshold of 300 mM (0.4944 ± 0.0311 mg·g^-1^), which was 124.7% higher than the non-inoculated group. Notably, both the inoculated and non-inoculated groups exhibited dual accumulation peaks at 100 mM and 300 mM, respectively. However, the standard error in the inoculated group at 300 mM was reduced, reflecting the ability of *P. indica* to enhance the stability of synthesis under high-salt conditions. At the same time, because this result is based on concentration data, the peak observed at 300 mM may indicate increased accumulation of liquiritin under high salinity, although this could reflect either enhanced biosynthesis or concentration effects due to reduced biomass. Future studies incorporating biomass-normalized metabolite analysis would help clarify this distinction.

#### 3.7.2 Dynamic response of glycyrrhizic acid accumulation

Similarly, the Two-way ANOVA for glycyrrhizic acid accumulation (**Table 5**) revealed significant main effects for both salt concentration (*P* = 0.023) and fungal colonization (*P* < 0.001), lacking a significant interaction (C × S: *P* = 0.448). This confirms a stable, additive accumulation pattern.

As shown in **Table 5**, the non-inoculated group exhibited a fluctuating adaptive response. A primary peak in glycyrrhizic acid accumulation occurred at 100 mM salt concentration (1.7200 ± 0.2093 mg·g^-1^), representing a 25.4% increase compared to the control. While relatively high levels were maintained at 300 mM (1.5900 ± 0.2118 mg·g^-1^), the content dropped significantly to 1.3100 ± 0.1097 mg·g^-1^ at 350 mM (a 23.8% decrease compared to the 100 mM peak).In contrast, *P. indica* induced a continuous enhancement pattern. glycyrrhizic acid accumulation in the inoculated group rose continuously across the 0–300 mM salt concentration range, peaking at 300 mM (2.5830 ± 0.1177 mg·g^-1^), which was 62.5% higher than the non-inoculated group. Even under high-intensity stress at 350 mM, the content in the inoculated group (1.8880 ± 0.1341 mg·g^-1^) remained significantly higher than in the non-inoculated group (+44.1%). Furthermore, the standard error was reduced, suggesting that *P. indica* may help mitigate the inhibitory effects of high salinity.

#### 3.7.3 Expression analysis of key genes in flavonoid and saponin biosynthesis

**Figure 4:**
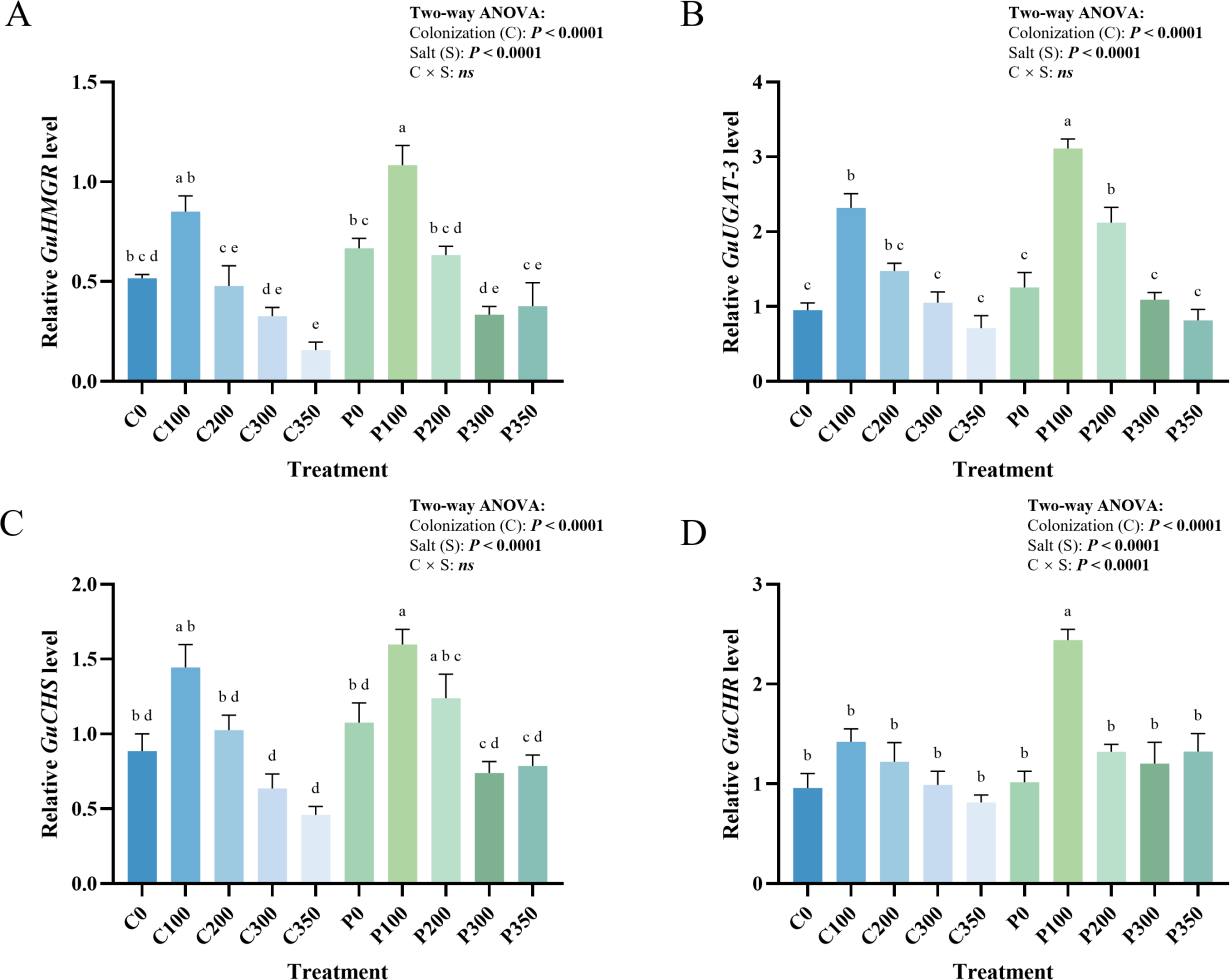
Modulation of key rate-limiting enzyme genes involved in the flavonoid and saponin biosynthetic pathways of (G) uralensis by (P) indica under salt stress. **(A)** GuHMGR expression; **(B)** GuUGAT-3 expression; **(C)** GuCHS expression; **(D)** GuCHR expression. Different lowercase letters above bars indicate significant differences among treatments at P < 0.05 according to Duncan’s multiple range test. Furthermore, the embedded text panels within the figures display the P-values derived from the Two-way ANOVA, which explicitly assesses the main effects of (P) indica colonization **(C)**, salt stress (S), and their interaction (C × S).

RT-qPCR analysis suggested that *P. indica* drives stress-adaptive synthesis by modulating genes encoding rate-limiting enzymes: *GuHMGR* (3-hydroxy-3-methylglutaryl-CoA reductase), *GuUGAT3* (UDP-glucuronosyltransferase 3), *GuCHS* (chalcone synthase), and *GuCHR* (chalcone reductase) (**Figure 4**) (Liu et al., 2024; Zhou et al., 2023). To rigorously evaluate this transcriptional regulation, a Two-way ANOVA was applied. In the uninoculated (C) group, the expression of the flavonoid pathway genes *GuCHS* (**Figure 4C**) and *GuCHR* (**Figure 4D**), along with the saponin-related *GuUGAT-3* (**Figure 4B**), showed a typical single-peak response, maximal at 100 mM. However, expression plummeted at 300–350 mM (dropping to 31.8%, 57.3%, and 30.8% of peak levels, respectively), confirming deep transcriptional inhibition by high salt stress.

*P. indica* colonization significantly reprogrammed these profiles. For *GuUGAT-3* and *GuCHS*, both colonization and salt stress independently and significantly enhanced expression, with no significant interaction (C × S: ns), indicating a robust additive regulatory effect. Under this additive regulation at 100 mM, it triggered an “early super-activation” where *GuUGAT-3* expression was 34.1% higher than in the control, reflecting a physiological pre-adaptation advantage. In contrast to the predominantly additive responses observed for most other variables, for the flavonoid synthesis gene *GuCHR*, a significant interaction between fungal colonization and salt stress was detected (C × S: *P* < 0.01). This interaction should be highlighted because it represents one of the clearest condition-specific responses in the present study. Notably, under extreme stress (350 mM), *GuCHR* expression remained 62.4% higher than in the non-inoculated group, indicating greater transcriptional stability under this specific stress condition.

#### 3.7.4 Specific modulation of the triterpenoid biosynthetic rate-limiting enzyme

For *GuHMGR* (**Figure 4A**), the key rate-limiting enzyme gene for triterpenoid saponin synthesis, Two-way ANOVA revealed highly significant main effects for both salt and colonization, but no significant interaction (C × S: ns), reflecting a stable additive modulation across the salt gradient. In the non-inoculated group, *GuHMGR* was highly sensitive to salt stress; expression increased transiently at 100 mM before declining continuously to 18.4% of peak levels at 350 mM.

*P. indica* remodeled this expression via a unique pre-activation and sustained maintenance pattern. At 100 mM, it induced pre-adaptive regulation, elevating expression by 27.3% over the group(C). Under extreme stress (350 mM), the fungus maintained *GuHMGR* transcriptional activity at a level 141.3% higher than in the non-inoculated group. These results indicate sustained expression of this key rate-limiting gene under severe stress; however, the term “pre-activation and sustained maintenance” is used here only as an interpretive summary and should not be taken as direct mechanistic proof. Overall, these results suggest that *P. indica* colonization and salt stress exert largely additive effects on the expression of key biosynthetic genes, with a clearer interaction effect observed only for *GuCHR*. This pattern points to a context-dependent physiological response under salt stress conditions, rather than a coordinated optimization mechanism.

## 4 Discussion

Current research predominantly relies on traditional inoculation methods, such as root irrigation with fungal suspensions or substrate mixing; however, inoculation techniques significantly influence colonization rates and growth-promoting efficacy. Furthermore, the species-specific compatibility between *G. uralensis* and *P. indica* remains insufficiently evaluated, and the mechanisms underlying their interaction are not yet fully understood. Additionally, although *P. indica* can be propagated *in vitro* (Liu et al., 2019), its compatibility and field persistence in major *G. uralensis* production regions lack empirical verification, restricting the realization of dual “ecological-economic” benefits (Guo, 2021).

This study indicates that *P. indica* may modify the salt response pattern of *G. uralensis*, potentially involving shifts in physiological responses under salt stress. Unlike the singular growth-promoting mechanism based on general SOD enhancement reported by Mao et al. (2016), or the broad-spectrum salt tolerance strategy involving a comprehensive increase in antioxidant enzyme activities observed in soybean by Chen and Mu (2023). *P. indica* exhibits a more refined, salt-concentration-dependent temporal regulation in *G. uralensis*, suggesting that the symbiotic system may contribute to redox balance through differential effects on antioxidant-related responses. This characteristic highlights significant interspecific variation; distinct from the comprehensive SOD activation observed in ryegrass (Wang, 2023), our findings add evidence to the view that plant responses to *P. indica* are species-dependent and should be interpreted in the context of the existing literature, rather than as a uniformly novel mechanism (Wang et al., 2024).

Within the 300 mM salt concentration window, the present data support the concurrent occurrence of growth maintenance, reduced oxidative damage, and increased medicinal compound accumulation; however, this pattern is better regarded as a descriptive integration of multiple responses than as direct evidence of a mechanistically demonstrated “triple-synergy mechanism.” The symbiosis maintains SOD functional stability (reducing activity variability by 73%) while significantly reducing MDA accumulation (12.6%) (**Figure 2**), concurrently driving a surge in the accumulation of liquiritin and glycyrrhizic acid (**Table 5**). Among these components, the reductions in MDA, the additive effects on antioxidant-related traits, and the increased accumulation of medicinal compounds are directly supported by the data, whereas the broader interpretation of coordinated metabolic optimization remains inferential. The pre-adaptive regulation under low-to-medium salt stress (< 300 mM) and the enhanced stability of the flavonoid terminal enzyme *GuCHR* under high salt stress (350 mM; expression upregulated by 9.4%) (**Figure 4**) may be consistent with a relatively efficient physiological response pattern, although direct evidence for metabolic cost was not obtained. By achieving a 12.6% reduction in membrane damage and elevating medicinal component levels with only 54.1% of the SOD activity input, *P. indica* may be associated with a physiological state in which antioxidant-related stress is alleviated and secondary metabolite accumulation is sustained. This finding distinguishes the mechanism from the singular activation of basal stress resistance genes reported in rice by Zhang et al. (2022).

Interestingly, the increase in shoot MDA (16.5%) was negatively correlated with the increase in SPAD (20.3%), indicating a potentially informative association between oxidative status and chlorophyll-related traits. Rather than being interpreted as evidence of enhanced photosynthetic function, the increase in SPAD may contribute to maintaining chlorophyll-related functionality under salt stress. In addition, this pattern may reflect temporal dynamics in oxidative responses, suggesting that initial colonization may transiently elevate oxidative stress, followed by improved regulation in later stages. However, because these observations are correlational, they should not be interpreted as evidence of a causal relationship between MDA accumulation and improved photosystem function or carbon assimilation.

*P. indica* appears to modulate the stress response of medicinal components in *G. uralensis* via salt-concentration threshold sensing and the targeted modulation of key biosynthetic enzyme genes. It maximizes the synthesis of liquiritin (bimodal activation) and glycyrrhizic acid (continuous accumulation) at 300 mM, and maintains a 44.1% advantage in glycyrrhizic acid accumulation even under high salt stress (350 mM), suggesting that *P. indica* may help alleviate the apparent trade-off between stress tolerance and medicinal component accumulation under salt stress. At the transcriptional level, these patterns are consistent with targeted changes in the expression of key biosynthetic genes: rather than indicating a generalized activation of all biosynthetic processes, *P. indica s*ynchronously upregulated specific key enzyme genes involved in flavonoid biosynthesis (*GuCHS*, *GuCHR*, *GuUGAT-3*) at 100 mM, increasing the expression of the key glycosylation enzyme gene *GuUGAT-3* by 34.1% (**Figure 4**). Conversely, it showed an early induction pattern at 100 mM and sustained expression under severe stress for the triterpenoid saponin rate-limiting enzyme gene *GuHMGR*, maintaining high expression levels (increased by 141.3%) even under extreme stress at 350 mM (**Figure 4**). This aligns with the multi-target regulatory characteristics of microorganisms described by Ma et al. (2024), while further clarifying the phasic nature of this regulation across a salt stress gradient. Simultaneously, *P. indica* significantly enhanced transcriptional stability under high salt conditions (reducing the standard error of *GuCHR* by 58.3%), thereby potentially contributing to greater stability in the expression of selected biosynthetic genes under high salt conditions. These results are consistent with the possibility that *P. indica* preferentially supports selected biosynthetic steps during stress-vulnerable stages, although direct evidence for resource allocation was not obtained in this study (He et al., 2022). This suggests that triterpenoid saponin biosynthesis in *G. uralensis* shows a stage-dependent response to salt stress.

Our data-driven analysis reveals that *P. indica* employs a highly dynamic, concentration-dependent regulatory strategy across the salt gradient, rather than a static defense mechanism. Under mild to moderate salinity (≤ 200 mM), the symbiont is associated with a relatively stable additive protective effect; it prioritizes membrane protection (MDA suppression) without redundantly over-activating antioxidant enzymes, which may be consistent with reduced physiological cost, although this was not directly measured in the present study. As osmotic pressure reaches the 300 mM threshold, a pronounced change in the response pattern is observed, coinciding with peak accumulation of key medicinal components (liquiritin and glycyrrhizic acid) to their absolute peaks. Crucially, under extreme stress (350 mM)—where non-inoculated plants suffer severe physiological collapse—interaction-associated effects become more evident in selected traits. This is characterized by a highly significant, time-specific reduction in the Senescence Index and the increased expression of terminal biosynthetic genes like *GuCHR*. Collectively, this gradient response suggests that the benefits of *P. indica* vary with stress intensity, ranging from predominantly additive protection under mild-to-moderate stress to interaction-associated effects in selected traits under extreme stress.

Furthermore, it is important to acknowledge the limitations regarding the extrapolation of pathway-level regulation. While our RT-qPCR data provide strong, targeted evidence for the symbiotic modulation of key rate-limiting enzyme genes (e.g., *GuHMGR* and *GuCHR*), evaluating a selected subset of genes does not fully capture the global reprogramming of the entire triterpenoid and flavonoid biosynthetic pathways. Accordingly, the mechanistic interpretations presented in the earlier sections should be understood within this limitation and should not be extended to the entire pathway without further evidence. Future investigations employing comprehensive multi-omics approaches—integrating whole-transcriptome sequencing and non-targeted metabolomics—are required to map the complete metabolic flux and fully elucidate the systemic regulatory networks driving this symbiotic enhancement.

## 5 Conclusion

In conclusion, *P. indica* significantly enhances the accumulation of liquiritin and glycyrrhizic acid while sustaining the salt stress adaptability of *G. uralensis*. These effects were associated with changes in ROS-related physiological indices and the upregulation of specific rate-limiting enzyme genes (e.g., *GuHMGR* and *GuCHR*), rather than with generalized activation of the entire biosynthetic pathway. In addition, the observed changes in SPAD and oxidative indicators suggest that *P. indica* may contribute to maintaining chlorophyll-related functionality under salt stress, although this should not be taken as direct evidence of enhanced photosynthetic function. The oxidative response also appears to show temporal variation, suggesting that initial colonization may transiently elevate oxidative stress, followed by improved regulation in later stages. These findings suggest that symbiotic microorganisms such as *P. indica* may help improve salt stress adaptation while supporting the accumulation of specific secondary metabolites through changes in antioxidant-related traits and the expression of selected biosynthetic genes, thereby contributing to improved salt stress tolerance and increased accumulation of major medicinal constituents. Consequently, this study offers a potential theoretical framework for the stress-resilient cultivation of medicinal plants.

## Data Availability

The original contributions presented in the study are included in the article. Additional raw data supporting the conclusions of this article are available from the corresponding author upon reasonable request, without undue reservation.
